# Novel Acetylcholinesterase Target Site for Malaria Mosquito Control

**DOI:** 10.1371/journal.pone.0000058

**Published:** 2006-12-20

**Authors:** Yuan-Ping Pang

**Affiliations:** Computer-Aided Molecular Design Laboratory, Mayo Clinic College of Medicine Rochester, Minnesota, United States of America; The Research Institute for Children, United States of America

## Abstract

Current anticholinesterase pesticides were developed during World War II and are toxic to mammals because they target a catalytic serine residue of acetylcholinesterases (AChEs) in insects and in mammals. A sequence analysis of AChEs from 73 species and a three-dimensional model of a malaria-carrying mosquito (*Anopheles gambiae*) AChE (*Ag*AChE) reported here show that C286 and R339 of *Ag*AChE are conserved at the opening of the active site of AChEs in 17 invertebrate and four insect species, respectively. Both residues are absent in the active site of AChEs of human, monkey, dog, cat, cattle, rabbit, rat, and mouse. The 17 invertebrates include house mosquito, Japanese encephalitis mosquito, African malaria mosquito, German cockroach, Florida lancelet, rice leaf beetle, African bollworm, beet armyworm, codling moth, diamondback moth, domestic silkworm, honey bee, oat or wheat aphid, the greenbug, melon or cotton aphid, green peach aphid, and English grain aphid. The four insects are house mosquito, Japanese encephalitis mosquito, African malaria mosquito, and German cockroach. The discovery of the two invertebrate-specific residues enables the development of effective and safer pesticides that target the residues present only in mosquito AChEs rather than the ubiquitous serine residue, thus potentially offering an effective control of mosquito-borne malaria. Anti-*Ag*AChE pesticides can be designed to interact with R339 and subsequently covalently bond to C286. Such pesticides would be toxic to mosquitoes but not to mammals.

## Introduction

Acetylcholinesterase (AChE), a serine hydrolase vital for regulating the neurotransmitter acetylcholine in mammals and in insects, has long been used as a target for pesticides. This enzyme has a deep and narrow active site, the bottom and opening regions of which are known as catalytic and peripheral sites, respectively [1], [2]. Current anticholinesterase pesticides for controlling pests, including African malaria-carrying mosquito (*Anopheles gambiae*), were developed during the World War II era. They react with a serine residue at the catalytic site, thus disabling the function of AChE. Because this serine residue is also present in mammalian AChEs, the use of these pesticides has been severely limited by their toxicity to mammals. Although it has long been assumed that humans are not harmed by low applications of the anticholinesterases as pests are more sensitive to the chemicals than humans, a recent report by the U.S. Environmental Protection Agency's Office of Inspector General indicates that some anticholinesterases can enter the brain of fetuses and young children and may destroy cells in the developing nervous system [3]. The use of anticholinesterase pesticides has also been limited by resistance problems caused by mosquitoes possessing AChE mutants such as the G119S mutant that is insusceptible to current pesticides [4].

Recent outbreaks of locally acquired mosquito-transmitted malaria in the United States demonstrate the continued risk for reintroduction of the disease [5]. To control mosquito-borne malaria through the use of effective and safer pesticides, this author has been searching for conserved target sites that are present only in mosquito AChEs. Such regions can be used as better target sites for design of new pesticides that would be devoid of the mammalian toxicity and the resistance problems of current pesticides. While a three-dimensional (3D) model of African malaria-carrying mosquito (*Anopheles gambiae*) AChE (*Ag*AChE) has been reported [6], no conserved and mosquito-specific region of *Ag*AChE has been reported until now. In this article, the author reports a sequence analysis of AChEs from 73 species that are currently available at the GenBank and a 3D model of *Ag*AChE generated by homology modeling and refinement with multiple molecular dynamics simulations performed on a terascale computer. These studies reveal two conserved residues (C286 and R339) present at the opening of the active site of *Ag*AChE but absent at those of mammalian AChEs.

## Results

### Homology model of *Ag*AChE

**Figure 1:**
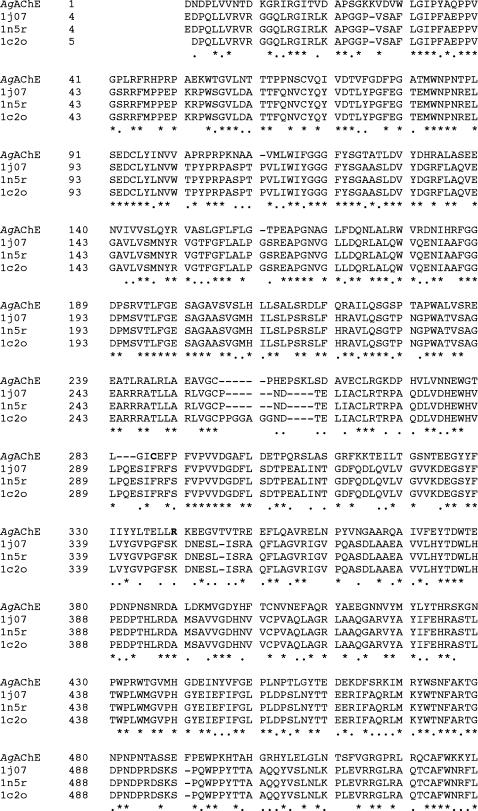
The SwissModel-generated multiple sequence alignments of *Anopheles gambiae* with mouse and electric eel acetylcholinesterases GenBank ID of the *A. gambiae* acetylcholinesterases sequence: BN000066; Protein Data Bank IDs of mouse acetylcholinesterase structures: 1J07 and 1N5R; Protein Data Bank ID of the electric eel acetylcholinesterase structure: 1C2O. The *A. gambiae*-specific residues (C286 and R339) are colored in red.

To search for a conserved and mosquito-specific region of *Ag*AChE, this author computationally determined a 3D model of a substrate-bound *Ag*AChE that is susceptible to current pesticides. The protein sequence of this AChE was obtained from GenBank (accession number: BN000066). A homology model of *Ag*AChE was first generated by the SWISS-MODEL program {http://swissmodel.expasy.org//SWISS-MODEL.html [7]} according to multiple sequence alignments using X-ray structures of two mouse and one electric eel AChEs as templates (Figure 1). The Protein Data Bank (PDB) IDs of the mouse AChEs are 1J07 and 1N5R [8]; the PDB ID of the electric eel AChE is 1C2O [9]. These crystal structures were automatically identified by the SWISS-MODEL program and have the highest sequence identity (46%) to *Ag*AChE. There were four regions of insertion and four regions of deletion in the *Ag*AChE sequence aligned with those of the crystal structures (Figure 1). In some regions of insertion and deletion, a proline residue, known as a helix breaker, is changed to other residues in *Ag*AChE. However, such changes do not affect the secondary structure of *Ag*AChE, because these regions do not adopt the helical conformation in the template structures (see secondary structures of the templates in Figure S1 of Supporting Information). The substrate-bound *Ag*AChE model was then built by manually docking acetylcholine into the active site of the homology model. The docking was guided by the substrate-bound *Torpedo* AChE (PDB ID: 2ACE [1]).

**Figure 2:**
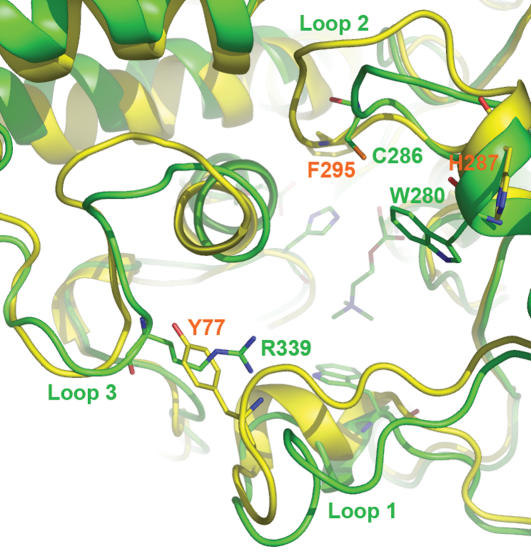
Overlay of *Anopheles gambiae* and human acetylcholinesterases *A. gambiae*: green; human: yellow; perspective: looking down onto substrate acetylcholine at the catalytic site.

The resulting *Ag*AChE complex model has nearly the same backbone conformation as those of the mouse and electric eel AChE structures except for residues 280–288 (loop 2) of *Ag*AChE (Figure 2), although many side-chain conformations of *Ag*AChE are different from the corresponding ones in the mouse and electric eel enzymes. Comparing to the corresponding region in the mouse and electric eel AChEs, loop 2 of *Ag*AChE is much shorter because it contains a region of deletion (Figure 1). Therefore, as part of the peripheral site, loop 2 of *Ag*AChE requires extensive refinement. At the opening of the active site of the unrefined *Ag*AChE complex model, the thiol group of C286 at loop 2 points away from W280 and Y333, thereby C286 does not interact with W280 and Y333; the guanidino group of R339 is not accessible to solvent as it is immediately surrounded by F75, F78, Y332, and W431.

### Refined model of *Ag*AChE

**Figure 3:**
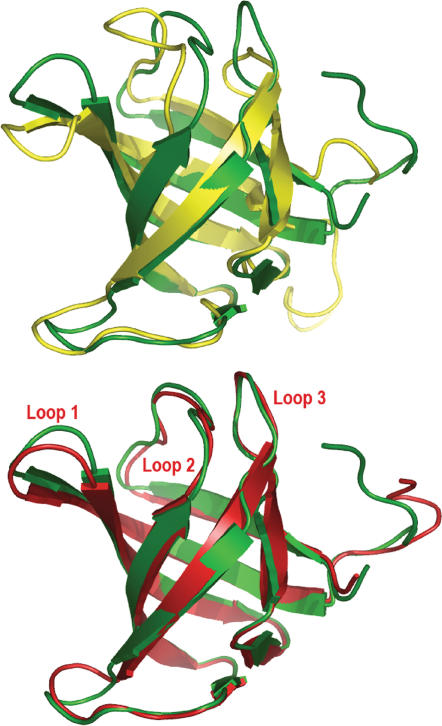
Overlays of a crystal structure with its unrefined and refined homology models The crystal structure: green, Protein Data Bank ID: 1XE1; the unrefined homology model: yellow, provided by the Protein Structure Prediction Centre (TMR01, http://predictioncenter.org/caspR/); the refined homology model: red, refined from TMR01 using the same protocol [15] for the 3D model of *Anopheles gambiae* acetylcholinesterase.

The homology complex model was then refined by multiple molecular dynamics simulations (MMDSs). The stochastic sampling of protein conformations achieved by MMDSs is more efficient than the sampling by a single long molecular dynamics simulation [10]–[15], and it is effective in refining loop conformations [15]. This MMDS refinement was validated through successful identification of small-molecule inhibitors of an MMDS-refined 3D model of a protease [15], [16]. The MMDS refinement method has proven successful in refining a homology model, provided by the Protein Structure Prediction Centre (TMR01, http://predictioncenter.org/caspR/), to a refined model that was nearly identical to the corresponding crystal structure (Protein Data Bank ID: 1XE1). Relative to the 1XE1 crystal structure, the alpha carbon root mean square deviation of the refined model was 1.7 Å, whereas the alpha carbon root mean square deviation of the homology model was 4.6 Å (Figure 3). The delta alpha carbon root mean square deviation for the MMDS-refined model is −2.9 Å, the best score for the Continuous CASP Model Refinement Experiment in 2006. The closeness in loops 1–3 between the refined model and the crystal structure (Figure 3) confirms the effectiveness of MMDSs in loop refinement. In the context of this advanced performance, MMDSs were used to refine *Ag*AChE, especially its loop 2 region.

In refining the homology model of the *Ag*AChE complex, 100 different molecular dynamics simulations (2.0 ns for each simulation with a 1.0-fs time step and with a different seed for starting velocity) were performed according to a published protocol [15]. An average of 50,000 trajectories of the complex obtained at 1.0-ps intervals during the last 500 ps of the 100 simulations was used as a refined 3D model of *Ag*AChE. The refined model was deposited to PDB on September 10, 2005 (PDB ID: 2AZG) and released at PDB on September 19, 2006.

**Figure 4:**
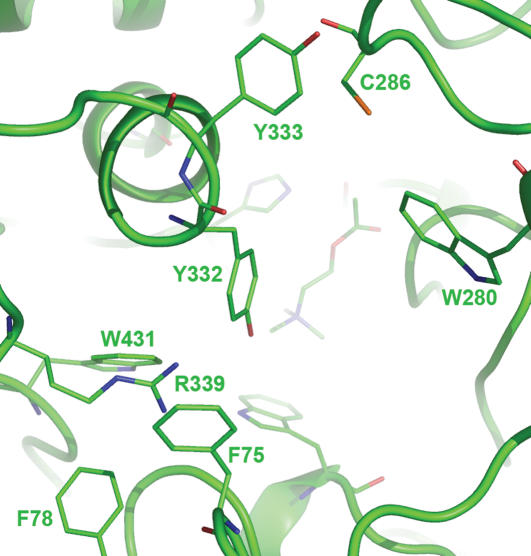
Close-up view of the peripheral site of *Anopheles gambiae* acetylcholinesterase Perspective: looking down onto substrate acetylcholine at the catalytic site.

Comparing to the unrefined model and human AChE (hAChE), the refined model has different main-chain conformations in three adjacent loops of residues 70–77 (loop 1), 280–288 (loop 2), and 333–349 (loop 3). These loops comprise most of the peripheral site of AChE (Figure 2). In contrast to the unrefined model, the refined model has the thiol group of C286 interacting with W280 and Y333 via sulfur-aromatic interaction [17] and the guanidino group of R339 partially accessible to solvent. The latter was caused by the side-chain conformational changes of F75 and Y332 and by the conformational change of loop 1 (Figure 4).

### Invertebrate-specific residues of AchE

**Figure 5:**
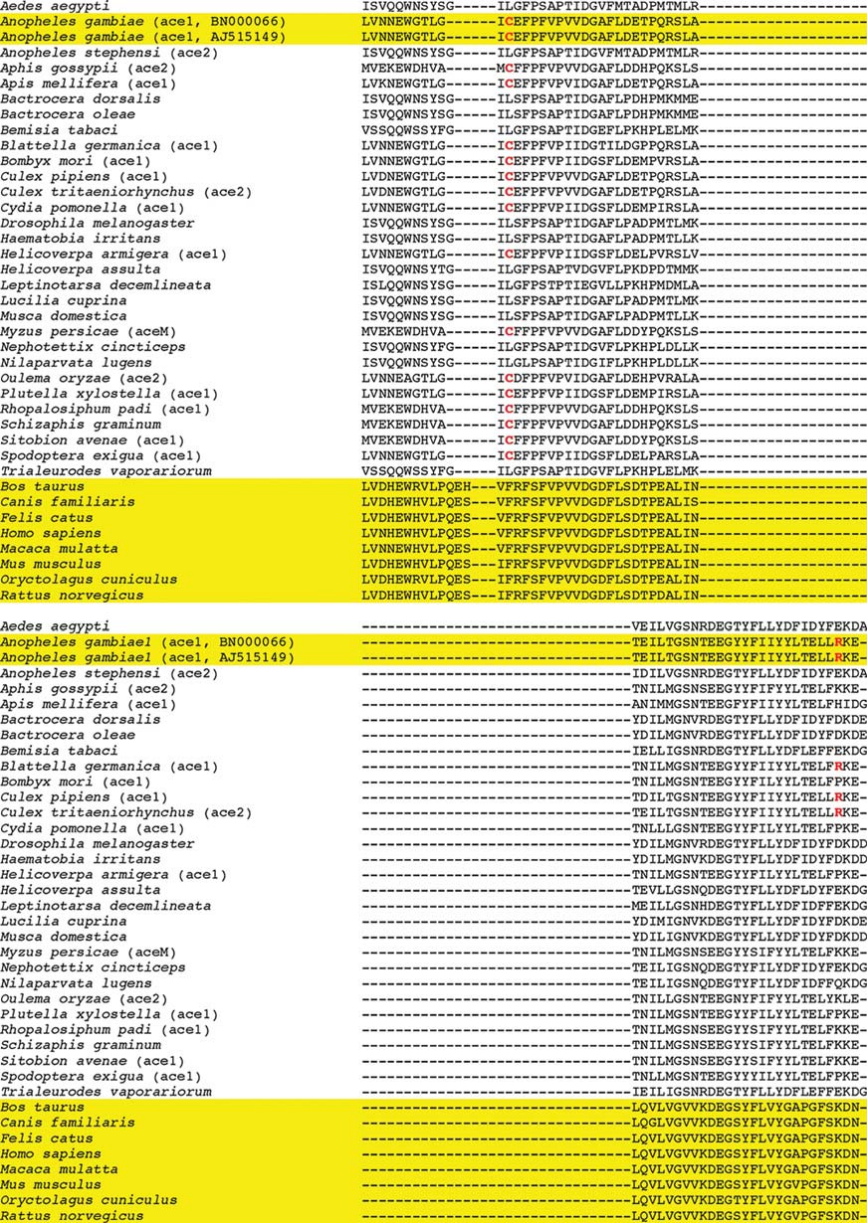
Multiple sequence alignments of acetylcholinesterases of insects and mammals listed in **Table 1**. The alignments were generated by CLUSTAL W (1.83). C286 and R339 of *Anopheles gambiae* acetylcholinesterase (AChE) and the corresponding residues in other species are colored in red. The mammalian and *A. gambiae* AChEs are highlighted in yellow. The multiple sequence alignments of AChEs of 73 species are shown in Figure S2 of Supporting Information.

Located at the peripheral site of the refined *Ag*AChE model, R339 has cation-pi interactions with F75, F78, Y332, and W431; this cationic residue stabilizes the aromatic residues that comprise part of the active site (Figure 4). The stabilizing role suggests that R339 is a conserved residue in mosquito AChEs. Interestingly, the residue corresponding to R339 of *Ag*AChE is absent in human AChE (hAChE); instead the phenol group of Y77 in hAChE occupies the region that corresponds to the region occupied by the guanidinium group of R339 (Figure 2). As shown in Figure 5 and Figure S2 of Supporting Information, using the CLUSTALW program [18], a sequence analysis of AChEs from 73 species (Table 1) that are currently available at the GenBank shows that R339 of *Ag*AChE is conserved in AChEs of only four insect species and absent in AChEs of all other species listed in Table 1. Of the 73 species, 30 and 8 of them are insects and mammals, respectively. The four insects are house mosquito (*Culex pipiens*), Japanese encephalitis-carrying mosquito (*Culex tritaeniorhynchus*), African malaria-carrying mosquito (*Anopheles gambiae*) including the one that is resistant to current pesticides (the G119S mutant, GenBank ID: AJ515149 [4]), and German cockroach (*Blattella germanica*).

**Table 1:**
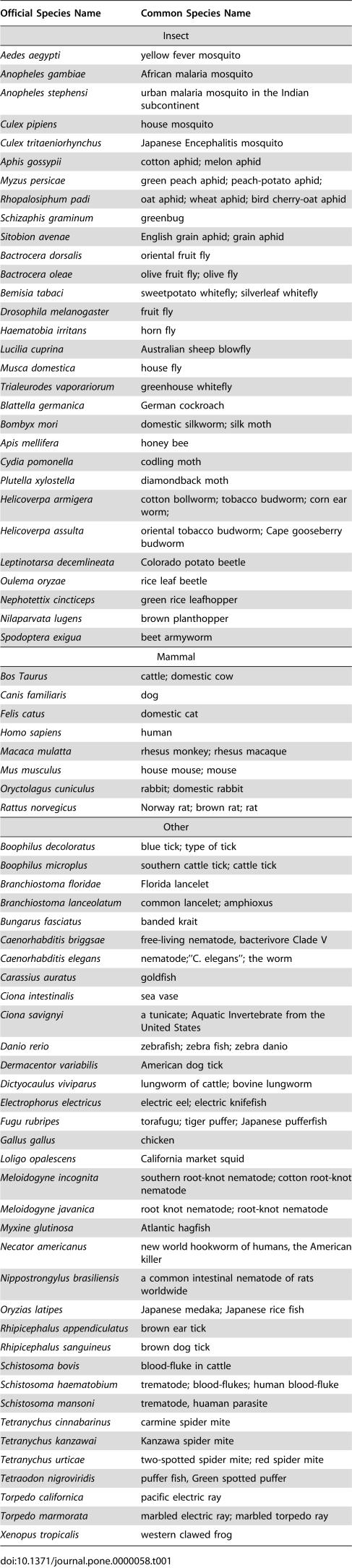
Official and Common Species Names of the 73 Acetylcholinesterases Used in This Study

Official Species Name	Common Species Name
Insect	
*Aedes aegypti*	yellow fever mosquito
*Anopheles gambiae*	African malaria mosquito
*Anopheles stephensi*	urban malaria mosquito in the Indian subcontinent
*Culex pipiens*	house mosquito
*Culex tritaeniorhynchus*	Japanese Encephalitis mosquito
*Aphis gossypii*	cotton aphid; melon aphid
*Myzus persicae*	green peach aphid; peach-potato aphid;
*Rhopalosiphum padi*	oat aphid; wheat aphid; bird cherry-oat aphid
*Schizaphis graminum*	greenbug
*Sitobion avenae*	English grain aphid; grain aphid
*Bactrocera dorsalis*	oriental fruit fly
*Bactrocera oleae*	olive fruit fly; olive fly
*Bemisia tabaci*	sweetpotato whitefly; silverleaf whitefly
*Drosophila melanogaster*	fruit fly
*Haematobia irritans*	horn fly
*Lucilia cuprina*	Australian sheep blowfly
*Musca domestica*	house fly
*Trialeurodes vaporariorum*	greenhouse whitefly
*Blattella germanica*	German cockroach
*Bombyx mori*	domestic silkworm; silk moth
*Apis mellifera*	honey bee
*Cydia pomonella*	codling moth
*Plutella xylostella*	diamondback moth
*Helicoverpa armigera*	cotton bollworm; tobacco budworm; corn ear worm;
*Helicoverpa assulta*	oriental tobacco budworm; Cape gooseberry budworm
*Leptinotarsa decemlineata*	Colorado potato beetle
*Oulema oryzae*	rice leaf beetle
*Nephotettix cincticeps*	green rice leafhopper
*Nilaparvata lugens*	brown planthopper
*Spodoptera exigua*	beet armyworm
Mammal	
*Bos Taurus*	cattle; domestic cow
*Canis familiaris*	dog
*Felis catus*	domestic cat
*Homo sapiens*	human
*Macaca mulatta*	rhesus monkey; rhesus macaque
*Mus musculus*	house mouse; mouse
*Oryctolagus cuniculus*	rabbit; domestic rabbit
*Rattus norvegicus*	Norway rat; brown rat; rat
Other	
*Boophilus decoloratus*	blue tick; type of tick
*Boophilus microplus*	southern cattle tick; cattle tick
*Branchiostoma floridae*	Florida lancelet
*Branchiostoma lanceolatum*	common lancelet; amphioxus
*Bungarus fasciatus*	banded krait
*Caenorhabditis briggsae*	free-living nematode, bacterivore Clade V
*Caenorhabditis elegans*	nematode;”C. elegans”; the worm
*Carassius auratus*	goldfish
*Ciona intestinalis*	sea vase
*Ciona savignyi*	a tunicate; Aquatic Invertebrate from the United States
*Danio rerio*	zebrafish; zebra fish; zebra danio
*Dermacentor variabilis*	American dog tick
*Dictyocaulus viviparus*	lungworm of cattle; bovine lungworm
*Electrophorus electricus*	electric eel; electric knifefish
*Fugu rubripes*	torafugu; tiger puffer; Japanese pufferfish
*Gallus gallus*	chicken
*Loligo opalescens*	California market squid
*Meloidogyne incognita*	southern root-knot nematode; cotton root-knot nematode
*Meloidogyne javanica*	root knot nematode; root-knot nematode
*Myxine glutinosa*	Atlantic hagfish
*Necator americanus*	new world hookworm of humans, the American killer
*Nippostrongylus brasiliensis*	a common intestinal nematode of rats worldwide
*Oryzias latipes*	Japanese medaka; Japanese rice fish
*Rhipicephalus appendiculatus*	brown ear tick
*Rhipicephalus sanguineus*	brown dog tick
*Schistosoma bovis*	blood-fluke in cattle
*Schistosoma haematobium*	trematode; blood-flukes; human blood-fluke
*Schistosoma mansoni*	trematode, huaman parasite
*Tetranychus cinnabarinus*	carmine spider mite
*Tetranychus kanzawai*	Kanzawa spider mite
*Tetranychus urticae*	two-spotted spider mite; red spider mite
*Tetraodon nigroviridis*	puffer fish, Green spotted puffer
*Torpedo californica*	pacific electric ray
*Torpedo marmorata*	marbled electric ray; marbled torpedo ray
*Xenopus tropicalis*	western clawed frog

Located on the opposite side of R339, C286 has favorable sulfur-aromatic interactions [17] with W280 and Y333 both located at the opening of the active site (Figure 4). In hAChE, the residue corresponding to C286 of *Ag*AChE is F295 that is located in the middle of the active site (Figure 2). The change of C286 to F295 in loop 2 has a large displacement (Figure 2); the distance between two alpha carbon atoms of C286 and F295 in an overlay of the two structures is 4.8 Å. As shown in Figure 5 and Figure S2 of Supporting Information, a sequence analysis of AChEs from the 73 species shows that C286 is present in AChEs of 17 invertebrate species and absent in AChEs of all other species listed in Table 1. The 17 invertebrates include house mosquito (*Culex pipiens*), Japanese encephalitis-carrying mosquito (*Culex tritaeniorhynchus*), African malaria-carrying mosquito (*Anopheles gambiae*) including the one that is resistant to current pesticides {GenBank ID: AJ515149 [4]}, German cockroach (*Blattella germanica*), Florida lancelet (*Branchiostoma floridae*), rice leaf beetle (*Oulema oryzae*), African bollworm (*Helicoverpa armigera*), beet armyworm (*Spodoptera exigua*), codling moth (*Cydia pomonella*), diamondback moth (*Plutella xylostella*), domestic silkworm (*Bombyx mori*), honey bee (*Apis mellifera*), oat or wheat aphid (*Rhopalosiphum padi*), the greenbug (*Schizaphis graminum*), melon or cotton aphid (*Aphis gossypii*), green peach aphid (*Myzus persicae*), and English grain aphid (*Sitobion avenae*).

## Discussion

### Novel acetylcholinesterase target site

**Figure 6:**
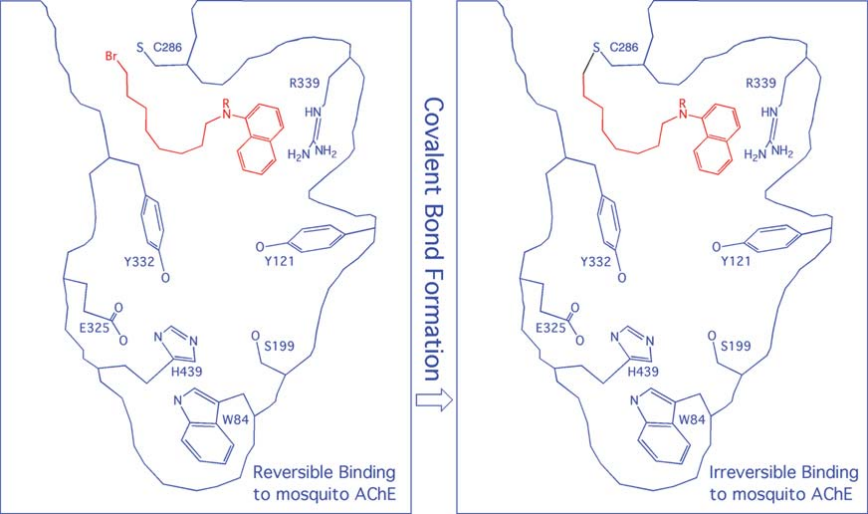
Cartoon representation of *Anopheles gambiae* acetylcholinesterase bound with a suicide inhibitor.

It has been reported that a native or engineered cysteine residue near the active site of an enzyme can hook a small molecule that binds, even loosely, at the active site, as long as the cysteine residue is able to react with an eletrophilic group of the molecule [19]. It has also been reported that reactive chemicals—which are covalently bonded to an engineered cysteine (H287C) at the peripheral site of mammalian AChEs (Figure 2)—are able to interfere with the substrate binding and subsequently inhibit the enzymes [20], [21]. Furthermore, it has been reported that, upon binding to the proximity of a native cysteine residue at the active site of a cysteine protease, a chemically stable molecule is able to bond covalently to the cysteine residue [22]. Based on these reports and on the proximity of C286 to its active site revealed by the 3D model of *Ag*AChE, it is conceivable that a chemically stable molecule can be made to react with C286 and irreversibly inhibit *Ag*AChE upon binding to the active site (Figure 6).

Virtual screening against the 3D model of *Ag*AChE using a published protocol [16], [23] has identified small molecules that have one functional group interacting with R339 and another functional group able to react with C286. Such molecules suggest a possibility of designing a small molecule that interacts simultaneously with C286 and R339, despite the average distance of the sulfur atom of C286 to the guanidino carbon atom of R339 is 13 Å. Because the guanidinium group of an arginine residue has multiple hydrogen bond donors and interacts favorably with aromatic groups, R339 can be used as an additional target site to facilitate the reaction of an inhibitor with C286 of *Ag*AChE. The unique presence of R339 and C286 in *Ag*AChE permits the design of a small molecule as a suicide inhibitor that first interacts with R339 leaving its electrophile in the proximity of C286 and then reacting with C286 (Figure 6), as illustrated by the example in reference 22.

### New pesticides to control malaria mosquitoes

Because of their species specificity demonstrated by the sequence analysis, C286 and R339 can be used as species markers for developing effective and safer pesticides that can covalently bond to C286 of *Ag*AChE. The absence of a cysteine residue in the peripheral site of mammalian AChEs means that pesticides targeting C286 and R339 would have less toxicity to mammals than current pesticides targeting the catalytic serine residue present in both mammals and insects. The aforementioned sequence analysis shows that both R339 and C286 are conserved in AChEs of African malaria-carrying mosquito (*Anopheles gambiae*), Japanese encephalitis-carrying mosquito (*Culex tritaeniorhynchus*), and house mosquito (*Culex pipiens*). The two residues are conserved also in the African malaria-carrying mosquito AChE mutant that is resistant to current pesticides [4]. It remains to be determined whether the two residues are conserved in AChEs of yellow fever mosquito (*Aedes aegypti*) and urban malaria-carrying mosquito in the Indian subcontinent (*Anopheles stephensi*), because the complete *ace1* sequences of *A. aegypti* and *A. stephensi* AChEs are currently unavailable. However, the above-described structural analysis shows that R339 interacts with F75, F78, Y332, and W431, and that C286 interacts with W280 and Y333. All these aromatic residues contribute importantly to the aromaticity of the active site of AChE that is required to bind its cationic substrate; R339 and C296 play a role in stabilizing these aromatic residues and conceivably have low mutation rates. Therefore, pesticides targeting R339 and C296 of *Ag*AChE would be devoid of the mammalian toxicity and the resistance problems of current pesticides.

It is certainly necessary to experimentally confirm the superiority of suicide inhibitors of *Ag*AChE as effective pesticides for the malaria mosquito control in terms of the toxicity and resistance issues. The results described here suggest a conceptually new paradigm for pesticide design, thus potentially offering an effective control of malaria mosquitoes. An effort to develop suicide inhibitors of *Ag*AChE is underway and will be reported in due course.

## Materials and Methods

### Homology modeling

The homology model of the apo *Ag*AChE was automatically generated by the SWISS-MODEL program available at http://swissmodel.expasy.org//SWISS-MODEL.html [7]. No manual adjustments were made to improve the multiple sequence alignments shown in Figure 1. The substrate-bound *Ag*AChE model was then built by manually docking acetylcholine into the active site of the homology model, guided by the substrate-bound *Torpedo* AChE (PDB ID: 2ACE [1]). The fully extended conformation of acetylcholine was used in the manual docking. The atomic charges of acetylcholine were obtained according to the RESP procedure [24] with an *ab initio* calculation at the HF/6-31G* level using the Gaussian98 program [25], and such charges are provided in Table S1 and Figure S3 of Supporting Information.

### Multiple molecular dynamics simulations

All MMDSs were performed according to a published protocol [15] using the SANDER module of the AMBER 8.0 program [26] with the Cornell et al. force field (parm96.dat) [27]. The topology and coordinate files used in the MMDSs were generated by the PREP, LINK, EDIT, and PARM modules of the AMBER 5.0 program [26]. All simulations used (1) a dielectric constant of 1.0; (2) the Berendsen coupling algorithm [28]; (3) a periodic boundary condition at a constant temperature of 300 K and a constant pressure of 1 atm with isotropic molecule-based scaling; (4) the Particle Mesh Ewald method to calculate long-range electrostatic interactions [29]; (5) iwrap = 1; (6) a time step of 1.0 fs; (7) the SHAKE-bond-length constraints applied to all the bonds involving the H atom; (8) default values of all other inputs of the SANDER module. The initial structure of the substrate-bound *Ag*AChE used in the MMDSs had no structural water molecules, and was solvated with 16,184 TIP3P water molecules [30] (EDIT input: NCUBE = 10, QH = 0.4170, DISO = 2.20, DISH = 2.00, CUTX = 8.0, CUTY = 8.0, and CUTZ = 8.0). The solvated *Ag*AChE complex system had a total of 56,926 atoms; it was first energy-minimized for 200 steps to remove close van der Waals contacts in the system, slowly heated to 300 K (10 K/ps), and then equilibrated for 1.5 ns. The energy minimization used the default method of AMBER 5.0 (10 cycles of the steepest descent method followed by the conjugate gradient method). The CARNAL module was used for geometric analysis and for obtaining the time-average structure. All MMDSs were performed on 200 Apple G5 processors dedicated to the Computer-Aided Molecular Design Laboratory.

## Supporting Information

Table S1 Amber Atom Types and Charges of Acetylcholine
(0.06 MB DOC)

Figure S1 The SwissModel-generated multiple sequence alignments and the secondary structure prediction of *Anopheles gambiae* acetylcholinesterase. GenBank ID of the * A. gambiae* acetylcholinesterase sequence: BN000066; Protein Data Bank IDs of mouse acetylcholinesterase structures: 1J07 and 1N5R; Protein Data Bank ID of the electric eel acetylcholinesterase structure: 1C2O. The *A. gambiae*-specific residues (C286 and R339) are colored in red.
(4.89 MB TIF)

Figure S2 Multiple sequence alignments of acetylcholinesterases of the 73 species listed in Table 1. The alignments were generated by CLUSTAL W (1.83). C286 and R339 of *Anopheles gambiae* acetylcholinesterase and the corresponding residues in other species are colored in red.
(10.24 MB DOC)

Figure S3 Definitions of atom names of acetylcholine
(1.11 MB TIF)
