# Supplementary material for: Novel Acetylcholinesterase Target Site for Malaria Mosquito Control
Source: PLoS One. 2006 Dec 20;1(1):e58. doi: 10.1371/journal.pone.0000058 (PMC1762403; doi:10.1371/journal.pone.0000058)
Supplement: Table S1 — Amber Atom Types and Charges of Acetylcholine (0.06 MB DOC) [file pone.0000058.s001.doc]

| Atom name* | Atom type | Charge |
| --- | --- | --- |
| C1 | CT | -0.327 |
| H1 | HC | 0.111 |
| H2 | HC | 0.111 |
| H3 | HC | 0.111 |
| C2 | C | 0.852 |
| O1 | O | -0.579 |
| O2 | OS | -0.493 |
| C6 | CT | 0.12 |
| H15 | H1 | 0.087 |
| H16 | H1 | 0.087 |
| C7 | CT | -0.053 |
| H13 | HP | 0.12 |
| H14 | HP | 0.12 |
| N1 | N3 | 0.043 |
| C4 | CT | -0.129 |
| H7 | HP | 0.119 |
| H8 | HP | 0.119 |
| H9 | HP | 0.119 |
| C5 | CT | -0.129 |
| H10 | HP | 0.119 |
| H11 | HP | 0.119 |
| H12 | HP | 0.119 |
| C3 | CT | -0.129 |
| H4 | HP | 0.119 |
| H5 | HP | 0.119 |
| H6 | HP | 0.119 |

*See Figure S3 for atom names.
