# Supplementary figures and images for: Novel Acetylcholinesterase Target Site for Malaria Mosquito Control
Source: PLoS One. 2006 Dec 20;1(1):e58. doi: 10.1371/journal.pone.0000058 (PMC1762403; doi:10.1371/journal.pone.0000058)

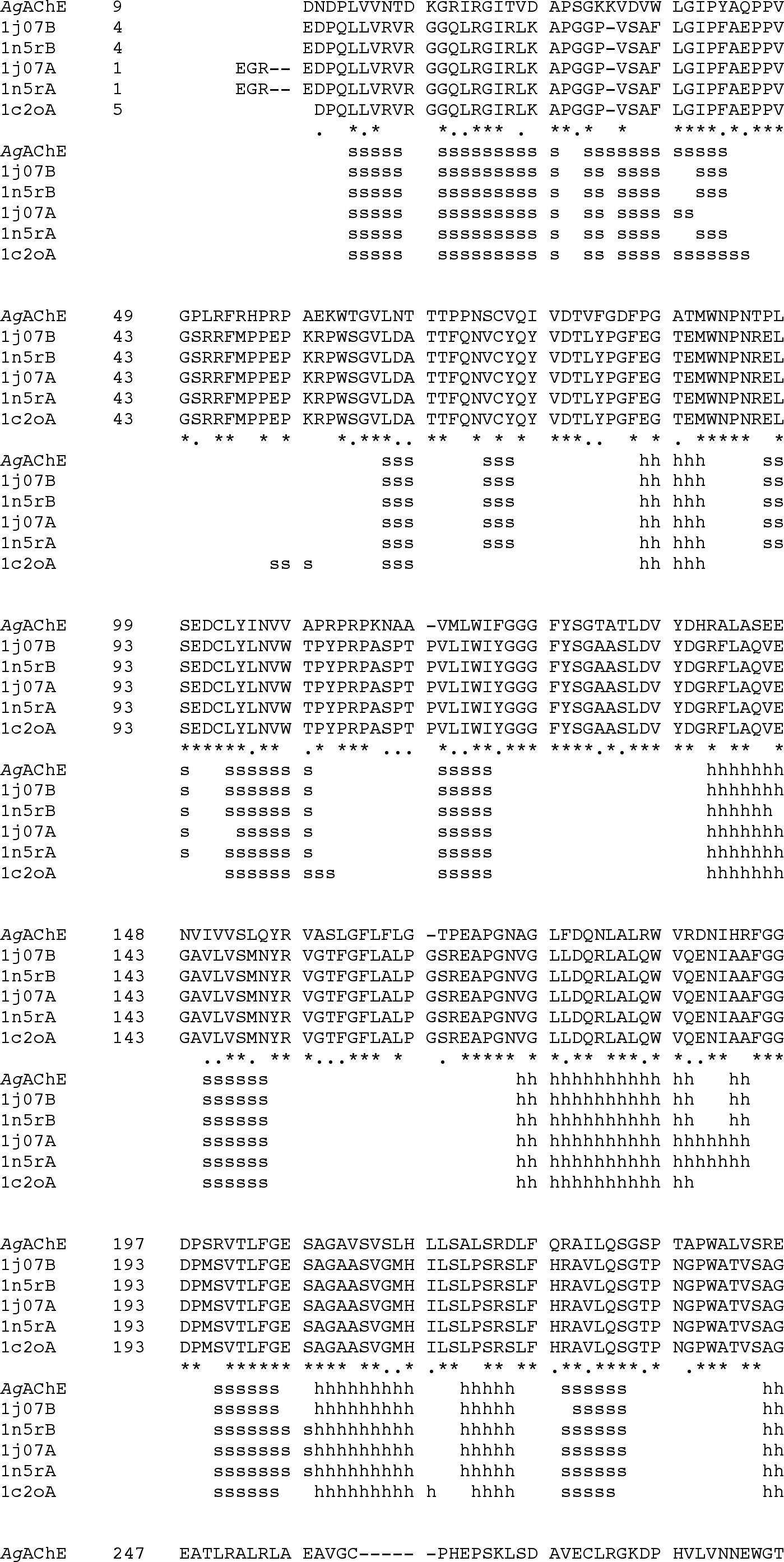

Supplement: Figure S1 — The SwissModel-generated multiple sequence alignments and the secondary structure prediction of Anopheles gambiae acetylcholinesterase. GenBank ID of the A. gambiae acetylcholinesterase sequence: BN000066; Protein Data Bank IDs of mouse acetylcholinesterase structures: 1J07 and 1N5R; Protein Data Bank ID of the electric eel acetylcholinesterase structure: 1C2O. The A. gambiae-specific residues (C286 and R339) are colored in red. (4.89 MB TIF) [file pone.0000058.s002.tif]

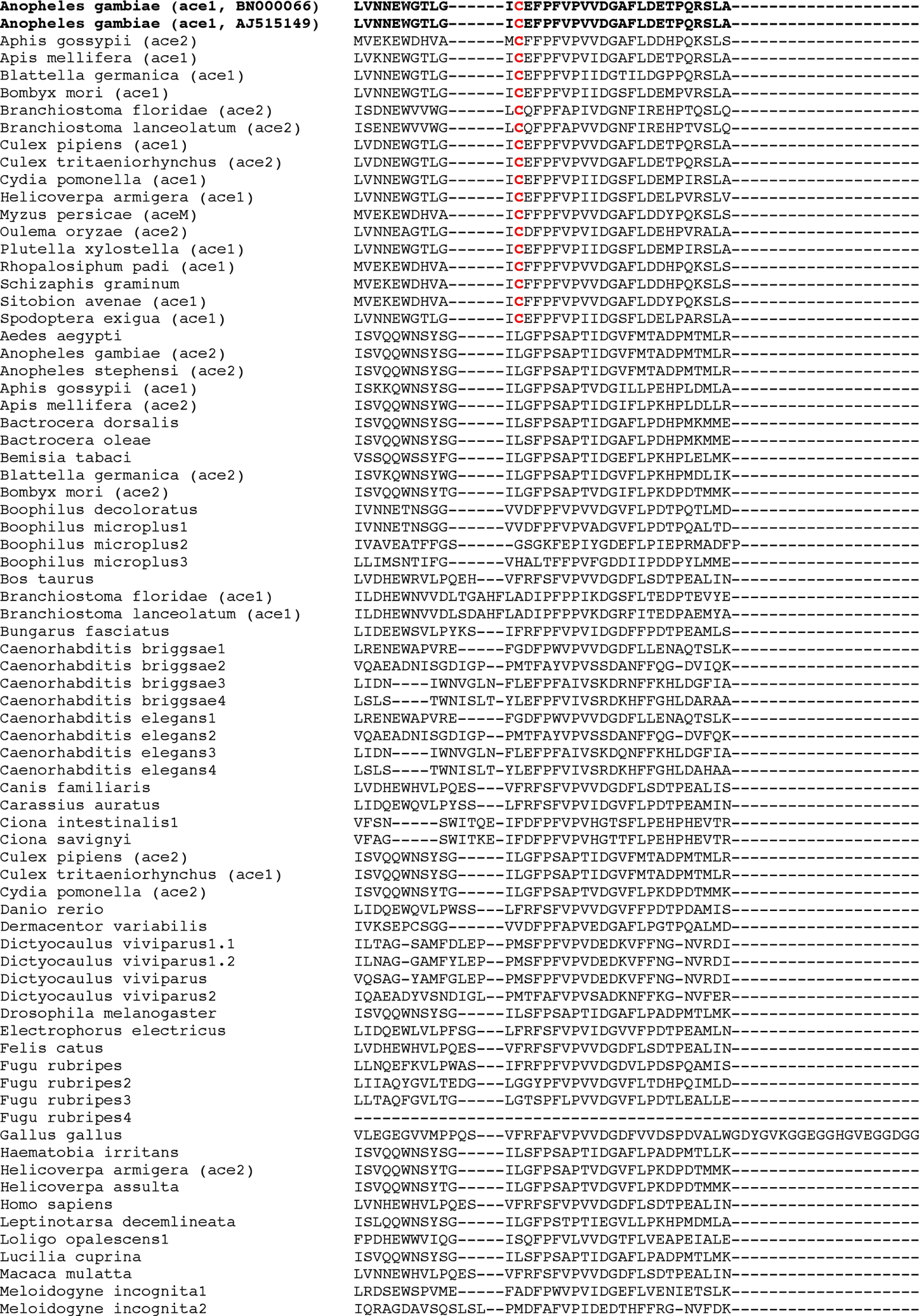

Supplement: Figure S2 — Multiple sequence alignments of acetylcholinesterases of the 73 species listed in Table 1. The alignments were generated by CLUSTAL W (1.83). C286 and R339 of Anopheles gambiae acetylcholinesterase and the corresponding residues in other species are colored in red. (10.24 MB DOC) [file pone.0000058.s003.doc]

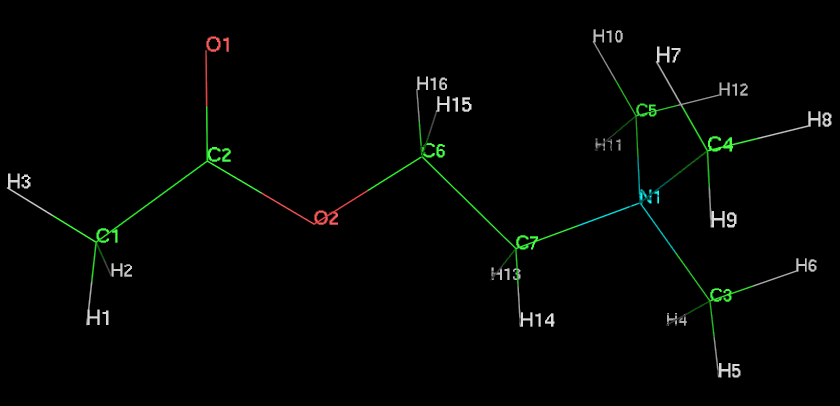

Supplement: Figure S3 — Definitions of atom names of acetylcholine (1.11 MB TIF) [file pone.0000058.s004.tif]
